# Optimized multiherbal combination and in vivo anti-skin aging potential: a randomized double blind placebo controlled study

**DOI:** 10.1038/s41598-023-32738-7

**Published:** 2023-04-06

**Authors:** Worrapan Poomanee, Nara Yaowiwat, Tunpidcha Pattarachaidaecharuch, Pimporn Leelapornpisid

**Affiliations:** 1 Department of Pharmaceutical Sciences, Faculty of Pharmacy, Chiang Mai University, Chiang Mai, 50200 Thailand; 2 Innovation Center for Holistic Health, Nutraceuticals, and Cosmeceuticals, Faculty of Pharmacy, Chiang Mai University, Chiang Mai, 50200 Thailand; 3 School of Cosmetic Science, Mae Fah Luang University, Chiang Rai, 57100 Thailand; 4 TP BEAUTY CARE Co., Ltd., Chiang Mai, 50300 Thailand

**Keywords:** Health care, Medical research, Signs and symptoms

## Abstract

The present study aimed to optimize a multi-herbal combination exerting the greatest antioxidant property using statistical method for anti-skin aging application as well as to elucidate its in vivo safety and anti-skin aging potential. The multi-herbal combination was optimized using a two-level, full factorial approach by exploring the correlation between the concentrations (0–3%w/v) of three extracts from *Centella asiatica* (CA)*, Momordica cochinchinensis* (MA), *Phyllanthus emblica* (EM). An anti-skin aging emulsion containing the optimized combination was then developed and evaluated for its physicochemical characteristics with its stability under storage conditions. The in vivo anti-skin aging potential of the emulsion was subsequently investigated among 60 women in a randomized, double-blind, placebo-controlled study. Skin hydration, elasticity and wrinkles at eye and cheek areas were measured at baseline, after 30 and 60 days of application. Before performance testing, in vivo skin irritation was evaluated using the patch test and homogeneity between groups was also statistically analyzed. According to the model describing the significant main effects of each extract and interaction effects between extracts on percent inhibition against DPPH radicals, the best multi-herbal combination consisted of 3%w/v EM and 3%w/v CA. The developed emulsion containing the combination presented smooth soft texture with good stability in terms of physical characteristics and biological property. Regarding the clinical study, no skin erythema and edema was reported among in all volunteers. After 60 days of application, significantly improved skin hydration, elasticity and wrinkles were observed in the test group. In addition, significantly reduced wrinkles were observed after 60 days in both skin areas of the test group. The anti-skin aging emulsion containing this optimized combination exhibited good safety and performance. Ultimately, this product comprises an effective anti-skin aging formulation for applications.

## Introduction

The cellular aging process occurs continuously over time after birth deteriorating not only biofunctions of internal organs but also physical appearances. Indeed, the aging sign of humans is markedly observed through aging skin appearances such as skin wrinkles, dry, sagging and rough skin that is principally attributed to both intrinsic and extrinsic factors generating mainly reactive oxygen species or free radicals^1^. Subsequently, a wide range of cellular degeneration related to oxidative stress is provoked including lipid peroxidative, damaged DNA, and inflammation by forming skin wrinkles as well as losing skin strength and integrity^2,3^. In addition, skin dark spots regarded as one of the skin aging manifestations is generally intensified by free radicals promoting skin melanogenesis and tyrosinase enzyme functions, a key enzyme of melanogenesis^4^. Together with the popularity of beautiful and young skin complexion, a range of anti-aging procedures have been extensively invented through the decades^5^. Natural anti-aging cosmetics is regarded as one of the fast-growing cosmetic markets across the globe. Some of the synthetic substances employed in cosmetics possibly provoke skin irritation and sensitivity especially in aged skin; thereby, highly promoting the requisition of using natural cosmetics. Nonetheless, to attain a successful and sustainable business in the natural cosmetics market, effective products, presenting multifunctional activities with innovations are mandatory^6^.

Thailand is regarded as one of the biodiverse regions of the world. A number of scientific research studies revealed that a vast diversity of Thai traditional herbs and plants exerted notable in vitro and in vivo anti-aging properties in several modes of action. *Centella asiatica* leaf extract has proven anti-aging potential with an outstanding moisturizing effect owing to its bioactive components including asiaticoside, madecassoside, asiatic acid and medecassic acid^7^. Haftek et al.^8^ reported that madecassoside derived from *C. asiatica* and vitamin C presented a synergistic anti-aging effect among human volunteers. Furthermore, 5% *C. asiatica* leaf extract-loaded cream exerted in vivo moisturizing and anti-inflammatory effects among 25 volunteers^9^. Another Thai anti-aging plant is *Momordica cochinchinensis* which could be used in fruit and peel extracts. *M. cochinchinensis* is a rich source of ß-carotenoids and lycopene, exerting strong antioxidant and skin whitening effects which are superior to vitamin C and E^10^. The clinical evaluation of Leevutinun et al.^10^ revealed that an antiwrinkle cream containing *M. cochinchinensis* extract significantly reduced skin roughness and increased skin hydration among 22 volunteers (ages 45 to 60) after 56 days of application. Furthermore, *Phyllanthus emblica* fruit extract has widely reported health benefits especially skin nourishing properties. Owing to its high vitamin C content together with high polyphenol contents, *P. emblica* extract exerted a strong inhibitory effect of tyrosinase enzyme, antioxidation, antibacterial, and anti-inflammatory effects. Regarding the anti-aging potential of *P. emblica* extract, matrix metalloproteinases (MMP)-2 and MMP-9 serving as degenerative enzymes of skin collagen and elastin could be attenuated by the extract^11^.

From the related literature, these three Thai herbal extracts were trusted to be promising active ingredients for anti-skin aging purposes. However, no evidence has proven the anti-aging combination effect of these three extracts which potentially promote anti-aging clinical outcome. As a result, the present study aimed to firstly optimize the multi-extract combination exerting a desirable antioxidant property by means of statistical method. An anti-aging emulsion containing the optimized multi-extract combination was subsequently developed and evaluated for its physical characteristics, stability as well as confirmed its in vivo anti-skin aging clinical efficacy by means of a randomized, double-blind, placebo-controlled trial conducting among 60 aged women. In addition, skin tolerance and safety profile of the product was investigated using the in vivo patch test among the volunteers.

## Materials and methods

### Plant extracts

Commercial herbal extracts including *Phyllanthus emblica* fruit extract (EM), *M. cochinchinensis* fruit extract (MA), and *C. asiatica* leaf extract (CA) dissolved in propylene glycol together with ingredients used in emulsions (cosmetic grade) were purchased and quality controlled by Namsiang by ArioMarketing Co., Ltd (Bangkok, Thailand). The contents of active components of CA which are asiaticoside and madecassicoside were controlled in range of 0.8–1.2%w/w. Polyphenol contents of EM and MA were more than 0.1%w/w. The manufacturing process of plant extracts complied with Act of legislation (Herbal product) 2019, Thai Food and Drug Administration to ensure that the process may comply with the IUCN Policy Statement on Research Involving Species at Risk of Extinction and the Convention on the Trade in Endangered Species of Wild Fauna and Flora.

### Chemicals

Absolute ethanol and deionized water were analytical grade. 1,1-diphenyl-2-picrylhydrazyl (DPPH) was purchased from Fluka (Buchs, Switzerland). Polyoxyethylene sorbitan monolaurate (Tween 20), sodium lauryl sulfate, l-ascorbic acid and Trolox were purchased from Sigma-Aldridge Inc. (Schnelldorf, Germany).

### Determination of in vitro antioxidant activity

DPPH radical scavenging assay following the method of Poomanee et al.^1^ was employed to identify the antioxidant capacity of the extracts, while Trolox and L-ascorbic acid served as positive controls. Briefly, each extract or positive control was diluted in ethanolic solution to produce five concentrations reacted with 120 mM DPPH in 1:10 ratio. The reaction was light-protected and left for 30 min. Afterwards, the absorbance was analyzed at 520 nm using a microplate reader (SpectroSTAR Nano®, Ortenberg, Germany). Percent inhibition and 50% inhibitory concentration (IC_50_) of the extract was calculated based on that of the negative control, namely absolute ethanol.

### Optimization of the multi-extract combination

The best extract combination showing the greatest antioxidant activity was optimized by 2-level full factorial design (FFD) owing to Design Expert Software Version 10.0.0 (Stat-Ease Inc., MN, USA). According to Table 1, percent of the extracts including EM (X_1_), MA (X_2_), and CA (X_3_) in the mixture were divided in two levels: low (− 1) and high (+ 1). Eight combinations were generated orthogonally (Table 2). No preservative and additive was added into the mixture to avoid interference effects. The extract mixture were dissolved in propylene glycol. The physical appearances of the extract mixtures were clear solution with light yellowish color. The experimental results were statistically analyzed by means of analysis of variance (ANOVA) to identify significant (*p* < 0.05) models, main effects, and interactions terms of the factors concerning antioxidant property of the extract. The final models were expressed in the Eq. (1) below.

1 $${\text{Y}} = {\upbeta }_{0} + \sum {{\upbeta }_{{\text{i}}} {\text{X}}_{{\text{i}}} } + \sum {{\upbeta }_{{{\text{ij}}}} {\text{X}}_{{1}} {\text{X}}_{{\text{j}}} } + \sum {{\upbeta }_{{{\text{ijk}}}} {\text{X}}_{{\text{i}}} {\text{X}}_{{\text{j}}} {\text{X}}_{{\text{k}}} }$$

where Y are the percent inhibitions on DPPH radicals. β_0_ is the average of the response data; β_i_, β_ij_, and β_ijk_ are regression coefficients of main effects, two-level interactions and three-level interaction, respectively. A desirable significant model with high coefficient of determination (R^2^) was obtained by excluding non-significant terms. Unless the interaction terms of the variables were significant (*p* < 0.05), the main effect was then kept in the final reduced model.

**Table 1:** Code levels and actual values of independent variables created according to the 2-level full factorial design (FFD); (%w/v).

Independent variables (X_i_)	Code levels and actual values	
	− 1	+ 1
% EM (X_1_)	0%	3%
% MA (X_2_)	0%	3%
% CA (X_3_)	0%	3%

**Table 2:** Experimental matrix generated by 2-level full factorial design and resulting data of dependent variables (Mean).

Run	Independent variables			Dependent variables
	%EM (X_1_)	%MA (X_2_)	%CA (X_3_)	% inhibition on DPPH radical (Y)
1	0	3	0	28.72
2	3	3	0	91.1
3	3	0	3	90.49
4	0	0	0	25.98
5	0	3	3	42.23
6	3	3	3	90.49
7	0	0	3	35.37
8	3	0	0	90.04

### Development of an anti-skin aging emulsion

An anti-skin aging emulsion, containing the optimized extract combination was developed as an emulsion. The aqueous phase consisted of water, glycerin, butylene glycol, sorbitol, caprylhydroxamic acid, 1,2-hexanediol, disodium EDTA, glyceryl stearate, ceteareth-20, and PEG-75 stearate whereas the oil phase consisted of dimethicone, petrolatum, *Argania spinosa* seed oil, *Rosa canina* L. seed oil, *Butyrospermum parkii* butter, cetearyl alcohol, tocopheryl acetate, and butylated hydroxytoluene. These two phases were separately heated using a water bath to reach 75 °C and subsequently combined using a high-speed homogenizer (8000 rpm) until homogenous. The obtained emulsion, which was then left under room temperature to reach 40 °C, was incorporated with the extract combination serving as test anti-skin aging emulsion. Physical characteristics of the formulation were evaluated in terms of viscosity and pH using a Brookfield rheometer (Model: R/S-CPS plate& plate, MA, US) and Apera pH testers (Apera instruments Co., Ltd., OH, USA). In addition, a texture analyzer (Model: TA. XTplusC, Stable Micro Systems, UK) was employed to determine cohesiveness and adhesiveness of the formulations compared with those of the emulsion base. Additonally, the emulsion base without the extract was assigned as a placebo for further clinical evaluation.

### Stability testing of the anti-skin aging emulsion

Stability profiles of the obtained emulsion containing the optimized extract combination and the emulsion base were evaluated in terms of physical stability and biological properties after storing under various conditions including room temperature with light (RTL) and without light (RTD), 4 °C, 45 °C for three months, and accelerated condition as 6 cycles of heating–cooling (HC). After storing, viscosity and pH value of each formulation were measured and compared with those initially. In addition, DPPH scavenging effect of the extract-loaded formulation was determined and compared with those initially. To evaluate the biological properties, 10% Tween 20 served as an extracting solvent for the extract-loaded formulation in a ratio of 1:1. The mixture was sonicated for 30 min and centrifuged at 10,000 rpm for 30 min (Model: Sorvall ST16R centrifuge, Thermo Fisher Scientific, Osterode am Harz, Germany). Supernatant was then collected and used for the evaluation.

### In vivo patch test

The patch irritation test based on good clinical practice was authorized by the Human Ethics Committee of the Faculty of Pharmacy, Chiang Mai University, Thailand (protocol no. 41/2020). Thirty healthy volunteers, having provided consent from all subjects, were included for this approach. As mentioned in the informed consent form, only summarized results and pictures of tested area which shown nothing attributed to participant’s identity were published. In addition, informed consent was obtained from all subjects or their legal guardians for both study participation and publication of identifying information/images in an online open-access publication. According to the European Society of Contact Dermatitis (ESCD) guidelines for diagnostic patch testing^12^, test product, placebo together with negative and positive controls (20 µL) filled separately onto the standard 8-mm Scanpor® Fin chamber (Dr. Ebeling & Assoc. GmbH, Hamburg, Germany), were applied on the clean upper back skin and left for 48 h. Bare skin served as a negative control, whereas 2% sodium lauryl sulfate was a positive control. After patch removal, erythema and edema reactions were observed at 24, 48 and 72 h and identified in terms of erythema and edema scores based on the Draize scoring system^13^. The primary irritation index (PII value) was then calculated following the method of Leelapornpisid et al.^14^.

### A randomized, double blind, placebo-controlled trial

Sixty healthy women aged 40 to 60 years were recruited in the clinical trial.

The inclusion criteria were shown as follows.

1. be able to inform consent
2. be able to follow the instruction of the research strictly
3. be 40—60 years old
4. be a woman
5. be healthy without any congenital skin disease.

The exclusion criteria were shown as follows.

1. be not able to inform consent
2. be not able to follow the instruction of the research strictly
3. have a congenital skin disease
4. have current skin diseases such as atopic dermatitis
5. be allergic to substances
6. be in a special treatment for any disease
7. have tattoos or wounds or skin disorders at the tested skin area
8. be a volunteer in other clinical research.

**Figure 1:**
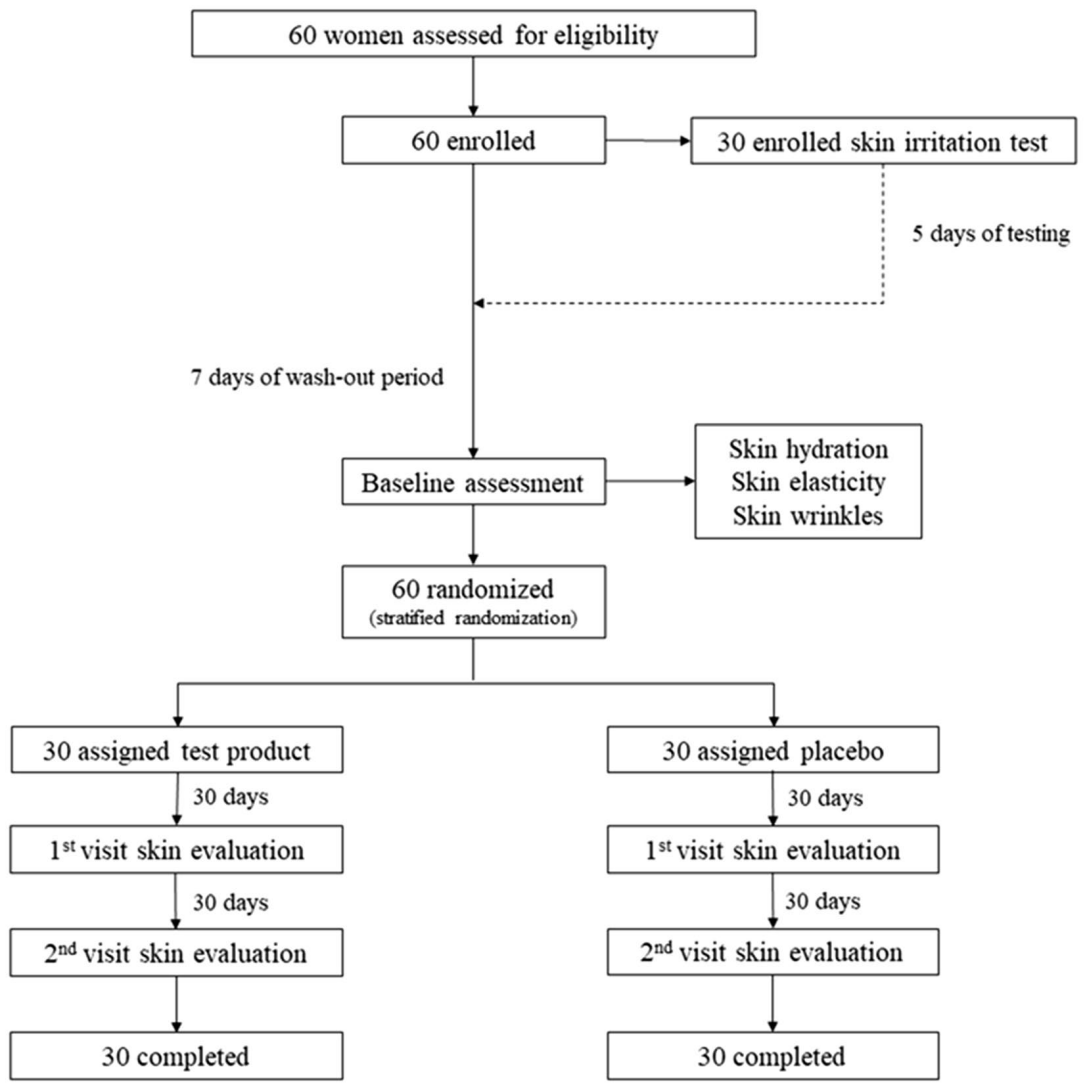
Overall process of the clinical study.

The Human Ethics Committee of the Faculty of Pharmacy, Chiang Mai University, Thailand approved the trial protocol (Protocol no. 41/2020) and all participants provided written, informed consent. The trial protocol was registered toward Thai Clinical Trials Registry (TCTR) on 17/01/2023 with a clinical trial registration number of TCTR20230117007. The methods for the randomized, double blind, placebo-controlled trial was carried out in accordance with the standard procedures of the Thai Guideline for Good Clinical Practice corresponding to ICH-GCP. The overall process is shown in Fig. 1. The participants were randomly divided in two groups as test (n = 30) and placebo (n = 30) groups based on stratified random sampling approach. The sample size was calculated and assigned following the method of Watson et al.^15^. According to the randomization, skin parameters related to skin aging including skin hydration, skin elasticity, and facial skin wrinkles of all participants were evaluated as baselines and sampled using stratified randomization. Moreover, prior homogeneity between test and placebo groups was statistically evaluated by means of an unpaired* t*-test^16^. Before baseline evaluation, participants were also requested to avoid using all skincare for 7 days (wash-out period). Regarding the blinding approach, test product and placebo were contained in identical packages with coded for each participant. Participants assigned as volunteer code numbers, product provider who gave the specific coded products to the participants and investigators who evaluate the skin parameters of the volunteers were blinded to the interventions. In addition, outcome assessors who conducted the randomization process, assigned volunteer codes, and product codes along with evaluate the results did not meet personally participant or know who the assigned volunteer of that volunteer code is. The participants were instructed to apply their assigned products on the entire face evenly twice daily for 60 days. To assess the compliance, three measures were executed. First method was checking the amount of product in two visits. Since the amount of product used per time was apparently informed to all participants at the first date. If the amount of product was reduced more than 50% at the first visit and almost finished at the second visit, the reduction of the product amount indicated the regularly use. Second method was assessing the clinical responses of each visit which apparently indicates the improvement of skin parameter after using the products. Third method was notifying frequently to all participants by the product provider to regularly use their products through the Line application – Chat group. Clinical assessments of the skin conditions were conducted at the corner of the eye and under the cheek bone after sample application at 30 and 60 days compared with those at baselines. Similar skin areas were measured in every period. Percent changes of the skin parameters were also calculated after 8 weeks. Before measuring, participants washed their entire face using Bioderma® Micellar water and waited 30 min in a climate-controlled room. All parameters were performed in triplicates.

Skin hydration was measured by Corneometer® CM825 (Courage and Khazaka Electronic GmbH, Cologne, Germany) and expressed in arbitrary units indicating the electrostatic capacity, proportional to the moisture content in the stratum corneum^16^.

Skin elasticity was measured by Cutometer® Dual MPA 580 (Courage and Khazaka Electronic GmbH, Cologne, Germany) that rendered a negative pressure of 450 mbar for 2 s and off pressure for 2 s in triplicates. The parameter of U_r_/U_f_ implying elastic recovery was evaluated herein using Cutometer MPA Software (Courage and Khazaka Electronic GmbH; Cologne, Germany).

Skin wrinkles were evaluated in terms of surface, volume, Ra, and Rz implying the amount of wavy surface compared with stretched areas, the virtual amount of liquid needed to fill in the skin furrow, the height of the skin furrow, and the arithmetic average of the skin roughness, respectively. Skin Visiometer® SV 700 (Courage and Khazaka Electronic GmbH; Cologne, Germany) was employed to directly visualize and analyze the skin wrinkles.

### Statistical analysis

Statistical analysis was carried out using SPSS 17.0 Software (IBM Co. Ltd., USA). All experiments were done in triplicates. All results were expressed as Mean ± SD. Statistical significance of the differences between biological properties of the extracts were evaluated using One-Way ANOVA with multiple comparison using Tukey. The paired *t*-test was employed to evaluate the stability testing. Prior homogeneity as well as the improvements between two participant groups were evaluated by independent *t*-test. The clinical improvement of skin conditions compared with the baselines of each group were analyzed using paired *t*-test and significant difference was defined at *p* < 0.05.

## Results and discussion

### Optimization of the Thai herbal extract combination

Three Thai herbal extracts, extensively regarded as anti-aging bioactive ingredients owing to their multifunctional activities, were systematically optimized to produce the greatest combinations inhibiting against free radicals. Free radicals are considered as crucial initiators of the skin aging process^3,17^. By virtue of abundant free radicals leading to oxidative stress, various skin deteriorations including skin inflammation, DNA damage as well as loss of skin structural components such as collagen, elastin, and hyaluronic acid were generated^2,3^. Herein, DPPH assay was employed since it potentially implied the results presenting a good correlation with phenolic and flavonoid bioactive compounds^18^ which were found in the extracts. The results revealed that EM exerted the strongest antioxidant effect with IC_50_ value of 0.56 ± 0.04 mg ml^−1^, whereas IC_50_ values of CA and MA were not detected since no concentration possibly inhibited the DPPH radicals by 50%. The IC_50_ values of positive controls as Trolox and L-ascorbic acid were 8.53 ± 0.15 µg ml^−1^ and 11.34 ± 0.11 µg ml^−1^.

**Figure 2:**
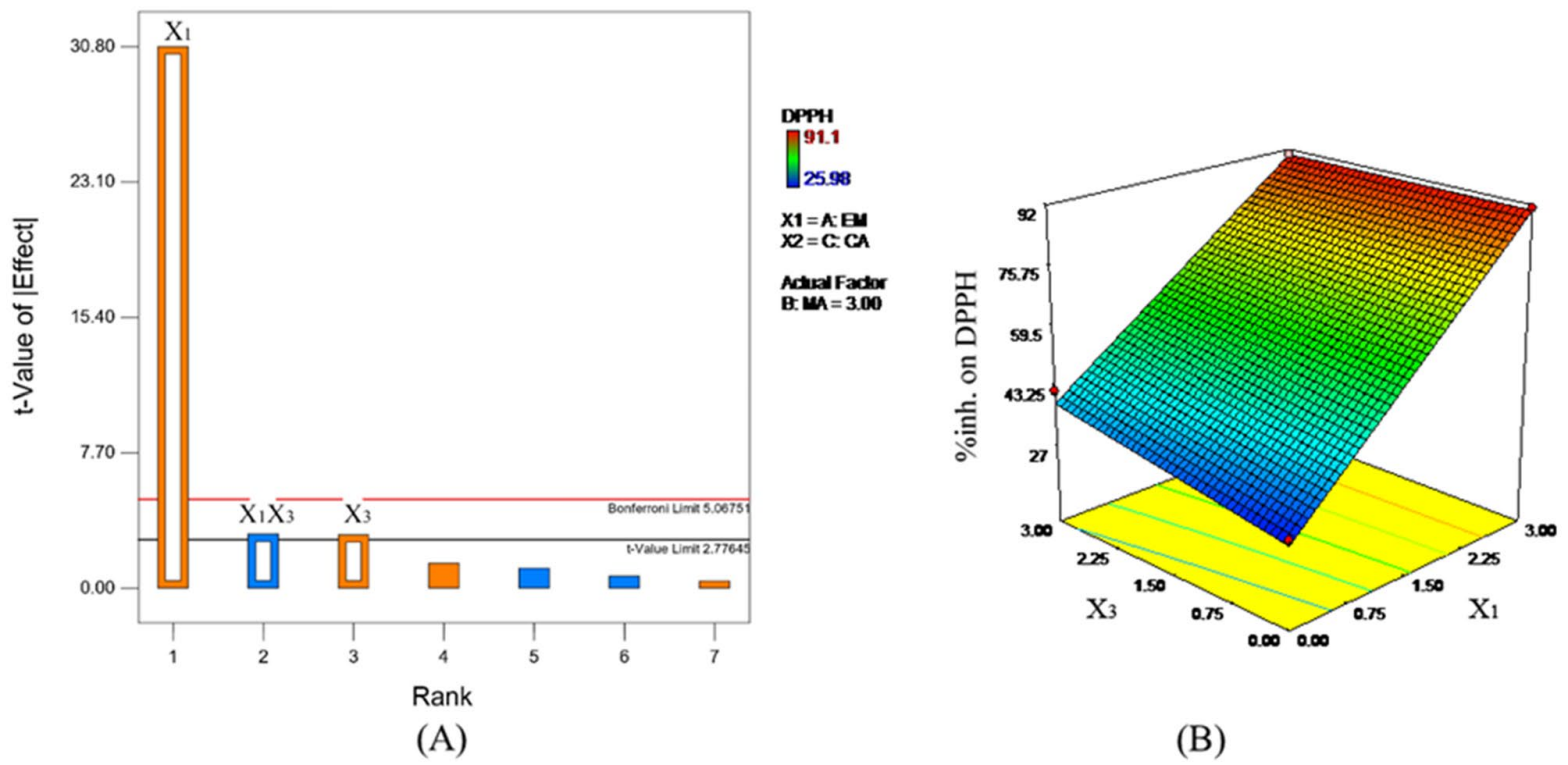
(**A**) Pareto charts illustrating absolute effects in a term of t-values on dependent variable as DPPH scavenging effect; (**B**) Response surface plots defining the combined effects of X_1_X_3_ on DPPH radicals in which factor X_2_ is 3%.

According to the in vitro antioxidant activity, the actual levels of all extracts based on FFD were established as shown in Table 1 so that desirable extract combinations exerting the greatest free radical scavenging effect were invented. Eight extract combinations showed multiple responses. The significant (*p* < 0.05) term was then selected based on absolute t-values shown in Fig. 2(A) to generate the significant (*p* < 0.05) final model presenting high coefficient of determination (R^2^) and high F-value. The influence of the three extracts on antioxidation of the extract combination were statistically evaluated and expressed in terms of the mathematic Eq. (2) below.

2 $$\% {\text{inhibition}}\,{\text{on}}\,{\text{DPPH}}\,\left( {\text{Y}} \right)\, = \,{61}.{8}0 + {28}.{\text{73X}}_{{1}} + {2}.{\text{84X}}_{{3}} {-}{2}.{\text{88X}}_{{1}} {\text{X}}_{{3}}$$

According to this significant model (*p* value < 0.0001, F value 322.41), about 99.59% of the percent DPPH inhibition could be interpreted precisely. EM (X_1_) exhibited the strongest positive influence on DPPH radical scavenging capacity as its highest regression coefficient. In addition, the main effect of CA (X_3_) significantly correlated positively to the response. Additonally, the significant interaction effect of EM (X_1_) and CA (X_3_) as shown in Fig. 2B illustrated that the greatest antioxidant property could be achieved apparently owing to the increase in EM. In the meantime, MA showed no significant influence on antioxidation. EM is a well-known bioactive resource providing a diversity of health benefits^19,20^. Owing to its high functional tannin contents, it highly exerted aesthetic utilities such as anti-aging, anti-tyrosinase, anti-inflammatory, and antimicrobial potentials. Besides, CA has been regarded as a potential anti-aging bioactive extract because of its strong in vitro and in vivo collagen-stimulating effect with low toxicity^7^. Therefore, EM played a pivotal role in the antioxidant effect of the extract combination.

As a result, the best extract combination for further developing as an anti-skin aging formulation was the combination of 3% EM and 3% CA (a ratio of 1:1).

### Physical characterization of the anti-skin aging emulsion

The obtained facial emulsion base (CB) presented an opaque smooth soft texture. The formulation presented an occlusive effect on the skin contributed by petrolatum. After incorporating with the extract combination, the formulation (CE) turned into light yellow owing to the hue of EM and CA. Physical characteristics and biological properties of the extract-loaded emulsion are shown in Table 3. After loading the extract, no significant change was noted in cohesion, adhesion, and pH of the extract-loaded emulsion. However, the viscosity of CE significantly decreased. In addition, the release profile of CE revealed a burst release pattern of the phenolic compounds with more than 50% of phenolic release in first hour and nearly 90% of phenolic release in 8 h (Unpublished data).

**Table 3:** Physical characteristics and biological properties of the extract-loaded emulsion (CE) and emulsion base (CB).

Sample	%inhibition of DPPH	Cohesion (G)	Adhesion (G s)	Viscosity (Pas)	pH
CB	13.25 ± 1.56	1.09 ± 0.03	62.70 ± 1.03	17.99 ± 1.44	4.5
CE	66.67 ± 7.31*	1.06 ± 0.01	61.87 ± 2.38	11.41 ± 0.63*	4.5

CB is the emulsion base; CE is the extract-loaded emulsion. The results were expressed in Mean ± SD.

*indicates statistical differences between tested parameters of CB and CE.

### Stability profiles of the anti-skin aging emulsion

**Figure 3:**
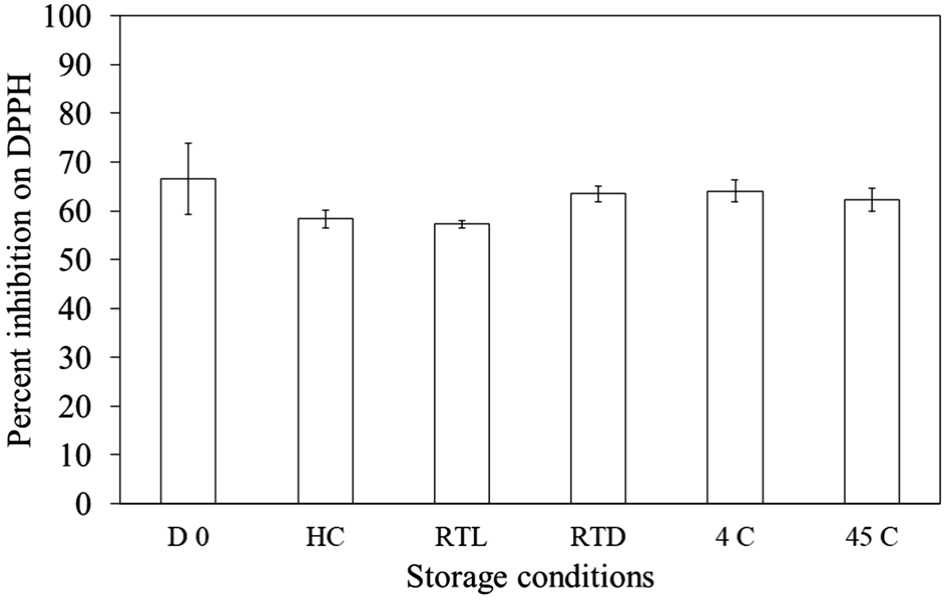
DPPH scavenging capacities of the extract-loaded emulsion (CE) at the initial and after storage under various conditions; 6 cycles of HC, RTL, RTD, 4 °C, and 45 °C.

After storing under various conditions, the physical appearances of both CE and CB presented no phase separation or pH change. In addition, viscosities of CE and CB were not significantly changed. However, the color of CE was slightly intensified after storing under 45 °C. Figure 3 illustrates that the DPPH radical scavenging effects of CE after stability testing were not significantly changed compared with those initially.

### In vivo skin irritation test

After fin chamber removal for 1 h, a slight erythema was shown at an area of 2% SLS among most of the volunteers. On the other hand, no erythema or edema was not observed in areas of CE and CB. PII values of bare skin, 2% SLS, CE, and CB were 0.05, 0.70, 0, and 0.01, respectively. This finding could confirm a good safety profile of these two formulations for human skin.

### In vivo anti-skin aging properties of emulsion containing optimized extract combination

#### Baseline characteristics

Sixty women volunteers included in this study were randomized in two groups as test (30 women, average age 48.67 ± 5.66) and placebo groups (30 women, average age 48.90 ± 5.82). According to the evaluation of prior homogeneity, no significant difference was observed between skin parameters (hydration, elasticity, and wrinkles in terms of surface, volume, Ra, and Rz) of test and placebo groups at baseline (*p* > 0.05) as shown in Table 4.

**Table 4:** Statistical evaluation between test and placebo groups prior to clinical trial.

Parameter	Site	Levene’s test for equality of variances		*t*-test for equality of means				
		F	Sig	t	df	Sig.^a^	Mean dif	Std. dif
Hydration	Eye	1.067	0.306	− 0.115	54.719	0.909	− 0.33667	2.93404
	Cheek	0.470	0.496	0.563	57.284	0.576	1.61333	2.86715
Elasticity	Eye	0.006	0.936	1.233	57.564	0.223	0.015800	0.012814
	Cheek	0.050	0.824	− 0.305	57.393	0.762	− 0.002617	0.008585
Skin wrinkle (Eye corner)	Surface	1.111	0.296	− 1.077	57.719	0.286	− 9.61800	8.93090
	Volume	0.789	0.378	0.527	51.309	0.601	2.85556	5.42044
	Ra	0.179	0.674	− 0.931	57.742	0.356	− 1.11111	1.19328
	Rz	1.723	0.194	− 0.919	55.160	0.362	− 0.25556	0.27805
Skin wrinkle (Cheek)	Surface	1.388	0.244	− 0.322	54.856	0.749	− 3.67633	11.43289
	Volume	4.553	0.037	1.634	48.524	0.109	5.91167	3.61784
	Ra	3.109	0.083	0.258	55.425	0.798	0.40000	1.55178
	Rz	1.134	0.291	0.701	55.533	0.487	0.27778	0.39654

^a^*p* > 0.05 indicating non-significant differences between test and placebo groups at baseline evaluated by independent* t*-test.

Mean dif. : Mean difference; Std. dif. : Standard error difference.

### In vivo anti-skin aging efficacy

**Figure 4:**
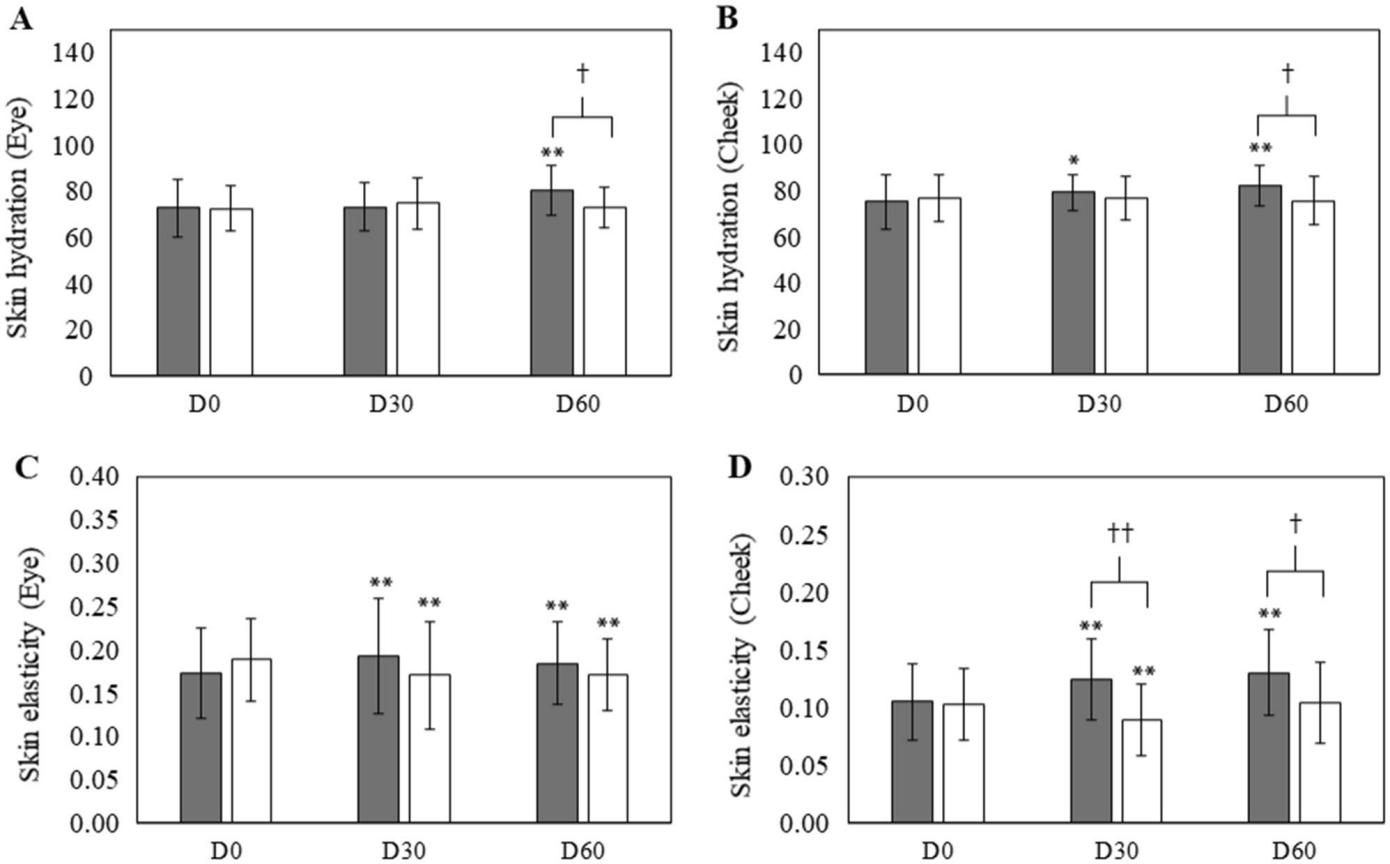
Skin hydration and skin elasticity measured at two skin sites, (**A**) the corner of eye; (**B**) under cheek bone and skin elasticity measured at two skin sites, (**C**) the corner of eye; (**D**) under cheek bone at baseline (D0), after 30 d (D30) and 60 d (D60) of application in the test () and placebo groups (). (Mean ± SD,*p < 0.05, **p < 0.001 vs. baseline; ^†^*p* < 0.05, ^††^*p* < 0.001 test vs. placebo group).

Skin hydration was represented in terms of skin water content herein measured by Corneometer. Figure 4A,B illustrate that extract-loaded emulsion effectively improved skin moisture at both eye and cheek areas after 60 days of application significantly differing from that of the emulsion base. At the cheek area, a significant increase in moisture could be observed after 30 days. Furthermore, improved skin hydration was shown in terms of percent changes of skin hydration from baseline^21^. In the case of the test group, percent change of skin hydration after 60 days of applying to eye and cheek areas were + 12.16% and + 10.89%, respectively, while percent change of skin hydration after using placebo at eye and cheek areas were + 2.30% and + 0.65%, respectively.

According to Fig. 4C,D, skin elasticities at eye and cheek areas were significantly (*p* < 0.001) increased in the test group compared with baseline, after 30 and 60 days while a significant decrease in skin elasticity at cheek area was observed in the placebo group after 30 days with percent reduction of -11.42% which was then up to + 2.10% after 60 days. No significant difference was observed between test and placebo groups at eye skin. Moreover, improved skin elasticity at the cheek area of the test group was significantly (*p* < 0.001) higher than that of the placebo group after 30 and 60 days of application. Compared with baseline, skin elasticities after 60 days of application of eye and cheek areas were + 9.02 and + 25.91%, respectively, in the test group.

**Figure 5:**
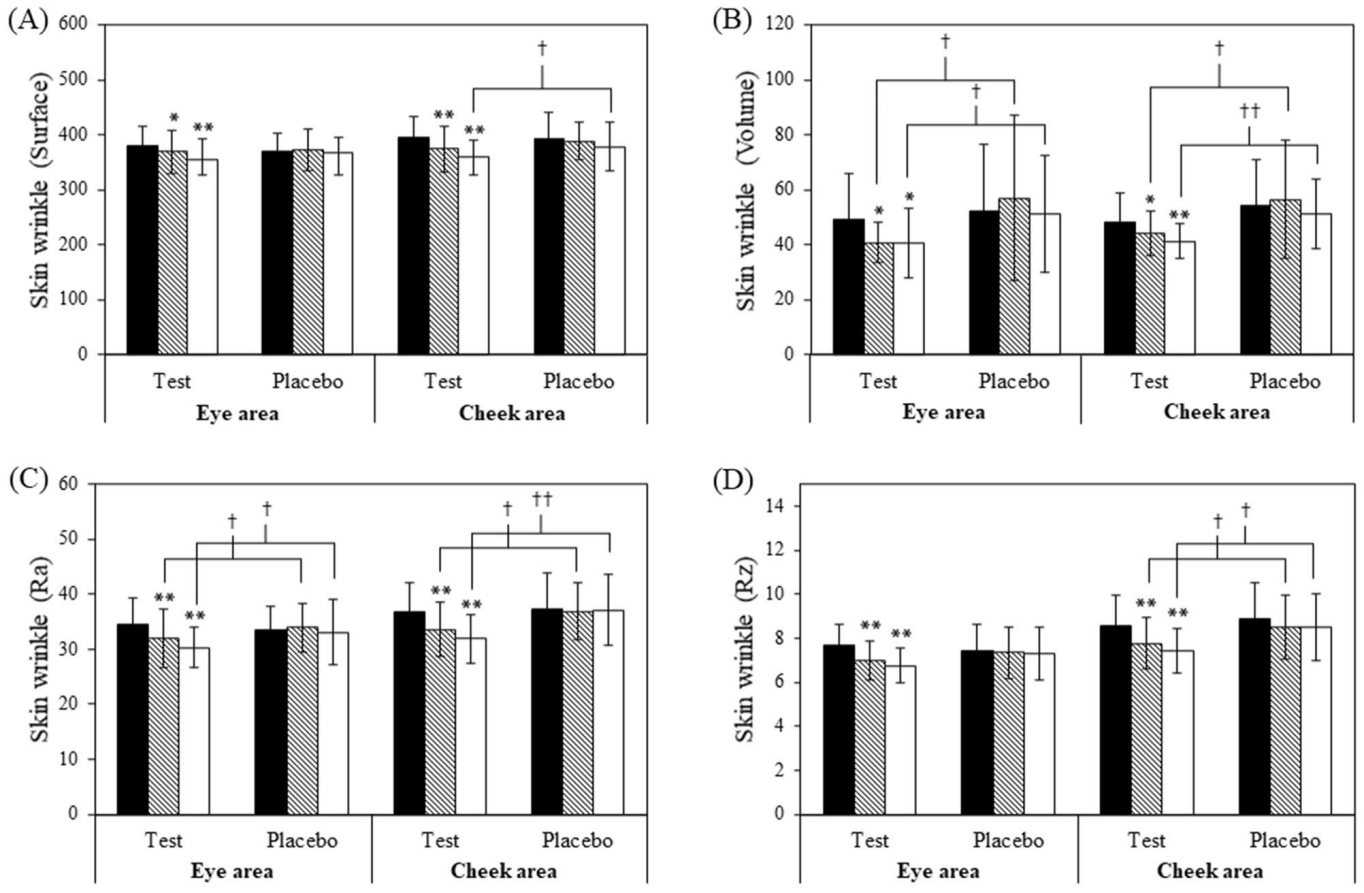
Skin wrinkle parameters, (**A**) surface; (**B**) Volume; (**C**) Ra; (D) Rz measured at two skin sites as the corner of eye and under cheek bone in the test and placebo groups at baseline (), after 30 d () and 60 d () of application. (Mean ± SD,*p < 0.05, **p < 0.001 vs. baseline; ^†^*p* < 0.05, ^††^*p* < 0.001 test vs. placebo group).

**Figure 6:**
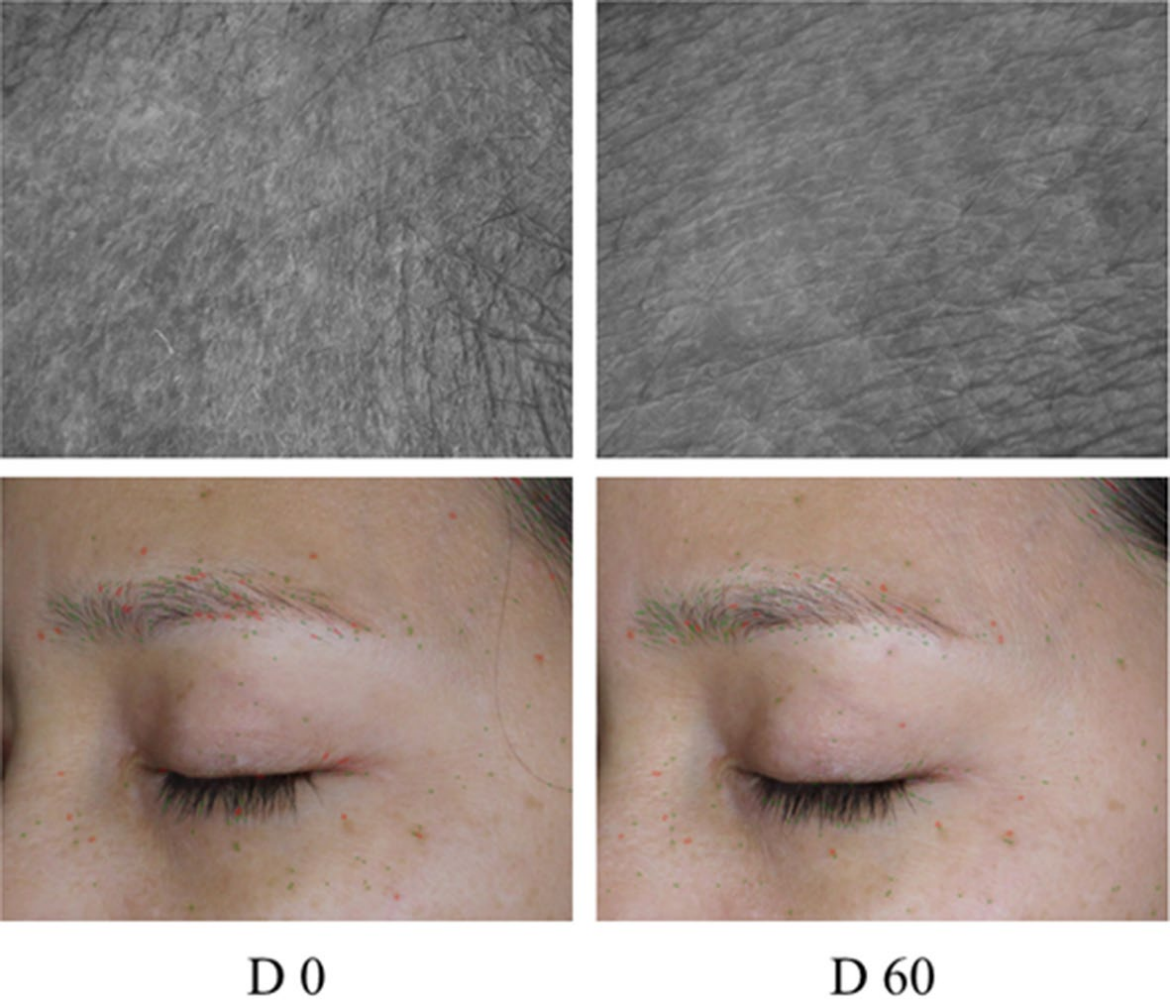
Images of skin wrinkle changes at the corner of the eye of one volunteer in test group after 60 d of application.

Aside from skin hydration and elasticity, four skin wrinkle and roughness parameters were employed to directly evaluate anti-skin aging including surface, volume, Ra and Rz. As shown in Fig. 5, significant improvements of all parameters from baseline were observed in the test group after 30 days of application. Comparing between test and placebo groups, significant differences were noted in surface at the cheek area after 60 days, in volume and Ra at both eye and cheek areas after 30 and 60 days as well as in Rz at the cheek area after 30 and 60 days as shown in Fig. 5. By two months, percent reduction of skin wrinkles in the test group at the eye area were − 6.05% (surface), − 12.69% (volume), − 11.27% (Ra), and − 11.29% (Rz). Additionally, those of wrinkle parameters at the cheek area were − 8.72% (surface), − 13.89% (volume), − 12.75% (Ra), and − 12.75% (Rz). In addition, image visualized using Visioscan (Fig. 6) illustrated the apparently improved skin wrinkles in eye areas of the test group. In case of placebo group, percent changes of skin wrinkles at cheek area after 60 days were − 2.75% (surface), − 2.25% (volume), 0.28% (Ra), and − 3.16% (Rz) while those at eye area after 60 days were − 0.68% (surface), 0.78% (volume), − 1.23% (Ra), and − 1.29 (Rz). The reduction in skin wrinkles of placebo group implied a moisturizing and anti-wrinkle capacities of the emulsion base which were apparently augmented through the addition of the multiherbal extract.

Owing to skin aging, extracellular connective tissue components composed mainly of collagen, elastin, and hyaluronic acid are degraded resulting in loss of skin elasticity, integrity, and formation of deep skin wrinkles^16^. Decrease in skin elasticity consequently caused skin sagging^5^. The degradation of these components is mainly attributed to cellular oxidative stress which potentiates a wide range of matrix metalloproteinase enzymes through the NF-kB and Activator protein-1 pathway^1,22^. In addition, elastase enzymes are induced by free radicals in both keratinocytes and fibroblasts^22^. As a result, the improved skin elasticity is certainly necessary for ameliorating skin wrinkles. Our result proved that the extracts exerted a notable anti-aging potential in terms of reducing skin wrinkles and improved skin elasticity among human volunteers. CA has been known for its outstanding skin aging properties attributed to its active component, madecassoside^8^. Aside from madecassoside, a triterpene compound, asiaticoside principally acts as a wound healing agent of CA through activating tumor growth factor ß (TGF-ß) and the SMAD signaling pathway rendering the induction of type I collagen synthesis^7^. Another extract in the combination is EM which has been considered as a potent antioxidant agent^11^. Due to its phytochemical compositions such as ascorbic acid, gallic acid, ellagic acid, EM showed notable anti-aging properties through different pathways including anti-tyrosinase effect and inhibitory effect on MMP-1, MMP-2, and elastase which are mainly responsible for degrading type I collagen in dermis, type VII collagen in the dermal–epidermal junction, and elastin, respectively^11^.

Another important physiological reaction contributing to skin aging is xerosis or dry skin^16^. Insufficient intercellular lipids together with alternation in intercellular lipid components during the aging process resulted in not only a weaker skin barrier but also a higher susceptibility to harmful environments^23^. In addition, an obvious occurrence of skin wrinkles is attributed to dry skin. As a result, antiwrinkle formulations exerting a notable moisturizing capacity are mandatory^16^. Our results imparted superior skin hydration effect of extract-loaded emulsion above those of emulsion base. Understandably, moisturizing formulations in the form of oil in water emulsion provide a moisturizing effect through three ingredients: skin occlusive materials, humectants, and emollients^24^. In addition to these ingredients, bioactive substances augmented skin moisturizing capacity to the formulation as presented in our result. The study of Ratz-Łyko et al.^9^ also reported that emulsion containing 5% *Centella asiatica* extract increased skin hydration by 25% among 25 human volunteers after 4 weeks of twice daily application which significantly differed from those emulsion base. Undoubtedly, CA provided improved skin hydration of the stratum corneum which was attributed to the presence of triterpene saponins with a hydrophilic sugar chain. This sugar substitute presents a water-binding capacity in nature offering higher skin water content detected in the uppermost skin layer^9^.

## Conclusion

Using a combination among active ingredients is currently ubiquitous in cosmetic products to enhance clinical efficacy and customer satisfaction. Our study firstly developed the anti-skin aging multi-herbal combination consisting of *Centella asiatica* leaf extract and *Phyllanthus emblica* fruit extract presenting outstanding antioxidant properties for anti-skin aging cosmeceutical products. The results from the randomized, double-blind, placebo-controlled trial demonstrated that the extract combination-loaded emulsion significantly improved skin hydration and skin elasticity contributing to apparently improved skin wrinkles after 60 days compared with those of emulsion base. Lastly, no volunteer showed skin irritation to the products. Ultimately, this product is effective for anti-skin aging applications.

## Data Availability

The datasets generated and/or analyzed during the current study are not publicly available due to the confidential dataset of clinical trial but are available from the corresponding author on reasonable request.

## References

[1] Poomanee W, Khunkitti W, Chaiyana W, Intasai N, Lin W, Lue S, Leelapornpisid P (2021). Multifunctional biological properties and phytochemical constituents of *Mangifera indica* L. seed kernel extract for preventing skin aging. Toxicol. Res..

[2] Zhang S, Duan E (2018). Fighting against skin aging: the way from bench to bedside. Cell. Transplant..

[3] Cao C, Xiao Z, Wu Y, Ge C (2020). Diet and skin aging-from the perspective of food nutrition. Nutrients.

[4] Manandhar B, Wagle A, Seong SH, Paudel P, Kim H, Jung H, Choi J (2019). Phlorotannins with potential anti-tyrosinase and antioxidant activity isolated from the marine seaweed *Ecklonia stolonifera*. Antioxidants.

[5] Ganceviciene R, Liakou AI, Theodoridis A, Makrantonaki E, Zouboulis CC (2012). Skin anti-aging strategies. Dermatoendocrinology.

[6] Emerald M, Emerald A, Emerald L, Kumar V (2016). Perspective of natural products in skincare. Pharm. Pharmacol. Int. J..

[7] Bylka W, Znajdek-Awizeñ P, Brzezinska M (2013). *Centella asiatica* in cosmetology. Adv. Dermatol. Allerg..

[8] Haftek M, Mac-Mary S, Le Bitoux M, Creidi P, Seité S, Rougier A (2000). Clinical, biometric and structural evaluation of the long-term effects of a topical treatment with ascorbic acid and madecassoside in photoaged human skin. Exp. Dermatol..

[9] Ratz-Łyko A, Arct J, Pytkowska K (2016). Moisturizing and antiinflammatory properties of cosmetic formulations containing *Centella asiatica* extract. Indian. J. Pharm. Sci..

[10] Leevutinun P, Krisadaphong P, Petsom A (2015). Clinical evaluation of Gac extract (*Momordica cochinchinensis*) in an antiwrinkle cream formulation. J. Cosmet. Sci..

[11] Pientaweeratch S, Panapisal V, Tansirikongkol A (2016). Antioxidant, anti-collagenase and anti-elastase activities of *Phyllanthus emblica, Manilkara zapota* and silymarin: an *in vitro* comparative study for anti-aging application. Pharm. Biol..

[12] Johansen JD, Aalto-Korte K, Agner T, Andersen KE, Bircher A, Bruze M (2015). European Society of Contact Dermatitis guideline for diagnostic patchtesting—Recommendations on best practice. Contact Dermatitis.

[13] Farage MA, Maibach HI, Andersen KE, Lachapelle J, Kern P, Ryan C, Ely J, Kanti A (2011). Historical perspective on the use of visual grading scales in evaluating skin irritation and sensitization. Contact. Derm..

[14] Leelapornpisid P, Mungmai L, Sirithunyalug B, Jiranusornkul S, Peerapornpisal Y (2014). A novel moisturizer extracted from freshwater macroalga (*Rhizoclonium hieroglyphicum* (C. Agardh) Kützing) for skin care cosmetic. Chiang. Mai. J. Sci..

[15] Watson REB, Ogden S, Cotterell LF, Bowden JJ, Bastrilles JY, Long SP, Griffiths CEM (2009). A cosmetic ‘anti-ageing’ product improves photoaged skin: a double-blind, randomized controlled trial. Br. J. Dermatol..

[16] Lee DE, Huh C, Ra J, Choi I, Jeong J, Kim S (2015). Clinical evidence of effects of *Lactobacillus plantarum* HY7714 on skin aging: A randomized, double-blind, placebo-controlled study. J. Microbiol. Biotechnol..

[17] Masaki H (2010). Role of antioxidants in the skin: anti-aging effects. J. Dermatol. Sci..

[18] Sadeer NB, Montesano D, Albrizio S, Zengin G, Mahomoodally MF (2020). The versatility of antioxidant assays in food science and safety-chemistry, applications, strengths, and limitations. Antioxidants..

[19] Kim JH, Yokozawa T, Kim HY, Tohda C, Rao TP, Juneja LR (2005). Influence of amla (*Emblica officinalis* Gaertn.) on hypercholesterolemia and lipid peroxidation in cholesterol-fed rats. J. Nutr. Sci. Vitaminol..

[20] Yokozawa T, Kim HY, Kim HJ, Tanaka T, Sugino H, Okubo T (2007). Amla (*Emblica officinalis* Gaertn.) attenuates age-related renal dysfunction by oxidative stress. J. Agric. Food. Chem..

[21] Manosroi J, Chankhampan C, Kitdamrongtham W, Zhang J, Abe M, Akihisa T (2020). In vivo anti-ageing activity of cream containing niosomes loaded with purple glutinous rice (*Oryza sativa* Linn.) extract. Int. J. Cos. Sci..

[22] Lim HY, Jeong D, Park SH, Shin KK, Hong YH, Kim E (2020). Antiwrinkle and antimelanogenesis effects of tyndallized *Lactobacillus acidophilus* KCCM12625P. Int. J. Mol. Sci..

[23] Duarte I, Hafner MS, Pedroso DMM, Silveira JE, Toyota R (2017). Sensitive skin: Review of an ascending concept. An. Bras. Dermatol..

[24] Lodén M (2005). The clinical benefit of moisturizers. J. Eur. Acad. Dermatol. Venereol..

